# Systematically Characterize the Anti-Alzheimer’s Disease Mechanism of Lignans from *S. chinensis* Based on In-Vivo Ingredient Analysis and Target-Network Pharmacology Strategy by UHPLC–Q-TOF-MS

**DOI:** 10.3390/molecules24071203

**Published:** 2019-03-27

**Authors:** Mengying Wei, Yuanyuan Liu, Zifeng Pi, Shizhe Li, Mingxin Hu, Yang He, Kexin Yue, Tianshu Liu, Zhiqiang Liu, Fengrui Song, Zhongying Liu

**Affiliations:** 1 Department of Pharmaceutical Analysis, School of Pharmaceutical Sciences, Jilin University, 1266 Fujin Road, Changchun 130021, China; 15584856155@163.com (M.W.); liuyuany16@mails.jlu.edu.cn (Y.L.); humx17@mails.jlu.edu.cn (M.H.); yuekxmails@163.com (K.Y.); tianshu421_liu@163.com (T.L.); 2 National Center for Mass Spectrometry in Changchun, Jilin Province Key Laboratory of Chinese Medicine Chemistry and Mass Spectrometry, Changchun Institute of Applied Chemistry, Chinese Academy of Sciences, Changchun 130022, China; mslab21@ciac.ac.cn (Z.P.); liuzq@ciac.ac.cn (Z.L.); songfr@ciac.ac.cn (F.S.); 3 Institute of Biomedical and Pharmaceutical Sciences, Guangdong University of Technology, Guangzhou 510006, China; lishizhe_2008@126.com; 4 Department of Pharmaceutical Analysis, School of Pharmacy and Food Science, Zhuhai College of Jilin University, 8 Anji East Road, Zhuhai 519041, China; heyang17@mails.jlu.edu.cn

**Keywords:** Alzheimer’s disease, lignan, metabolite identification, *Schisandra chinensis*, target-network pharmacology, UHPLC–Q-TOF-MS

## Abstract

Lignans from *Schisandra chinensis* (Turcz.) Baill can ameliorate cognitive impairment in animals with Alzheimer’s disease (AD). However, the metabolism of absorbed ingredients and the potential targets of the lignans from *S. chinensis* in animals with AD have not been systematically investigated. Therefore, for the first time, we performed an in-vivo ingredient analysis and implemented a target-network pharmacology strategy to assess the effects of lignans from *S. chinensis* in rats with AD. Ten absorbed prototype constituents and 39 metabolites were identified or tentatively characterized in the plasma of dosed rats with AD using ultra high-performance liquid chromatography coupled with quadrupole time-of-flight mass spectrometry. Based on the results of analysis of the effective constituents in vivo, the potential therapeutic mechanism of the effective constituents in the rats with AD was investigated using a target-network pharmacology approach and independent experimental validation. The results showed that the treatment effects of lignans from *S. chinensis* on cognitive impairment might involve the regulation of amyloid precursor protein metabolism, neurofibrillary tangles, neurotransmitter metabolism, inflammatory response, and antioxidant system. Overall, we identified the effective components of lignans in *S. chinensis* that can improve the cognitive impairment induced by AD and proposed potential therapeutic metabolic pathways. The results might serve as the basis for a fundamental strategy to explore effective therapeutic drugs to treat AD.

## 1. Introduction

Alzheimer’s disease (AD) has become one of the leading diseases affecting the health of the elderly population, with a prevalence of 5% after 65 years of age, which increases to approximately 30% in people aged 85 years or older [1]. It is characterized by progressive cognitive impairment, including impaired judgment, decision-making, and orientation; the disease is often accompanied by psycho–behavioral disturbances and language impairment in the later stages [2]. The pathogenesis of AD is still unclear. A widely accepted hypothesis is the amyloid cascade hypothesis, which suggests that amyloid beta (Aβ) deposition might trigger neuronal dysfunction and death in the brain, resulting in AD [3]. Alzheimer’s disease has imposed a heavy burden on the healthcare and socio-economic development of elderly population. Acetylcholinesterase inhibitors (AChEIs) and non-competitive *N*-methyl-d-aspartate antagonists are the first line drugs for treating AD. The therapeutic effects of AChEIs are attributable to the increase in acetylcholine (ACh) levels in the brain [4]. The non-competitive *N*-methyl-d-aspartate antagonist might protect neurons from glutamate-mediated excitotoxicity, and it has been reported to be beneficial in the advanced stages of AD [5]. These two kinds of drugs can slow the emergence of the behavioral and psychotic symptoms associated with AD. However, there is still no convincing evidence to suggest that these drugs can halt or reverse the progression of AD. Therefore, it is important to study the drugs that can effectively ameliorate the progression of AD.

For thousands of years, medicines have been closely linked to natural products, which are still a significant source of new drugs [6]. *Wuweizi*, in Chinese, is the dry fruit of *Schisandra chinensis* (Turcz.) Baill. (Magnoliaceae); it mainly contains lignans, sugars, tannins, essential oils, and organic acids [7]. The lignans isolated from *S. chinensis* have been associated with antihepatotoxic, antioxidant, detoxification, and neuroprotective effects, and these effects have potential roles in the treatment of AD [8,9]. Lignan extracts from *S. chinensis* can ameliorate cognitive impairment, and they have been shown to possess neuroprotective effects in animals with AD [10,11,12,13]. In our laboratory, we also proved that the extract of *S. chinensis* can significantly shorten the escape latency time and increase the crossing time of target and central zones (%) in rats with diabetic encephalopathy [14]. Studies analyzing the in vivo metabolism of lignans from *S. chinensis* have mainly been conducted in normal rats [15,16], and the possible in vivo metabolic pathways of these lignans have not been studied in rats with AD.

An organism is a complexly networked biological system. Several diseases (such as diabetes, depression, and cancer) are caused by altered expression of multiple genes or proteins. Therefore, drugs designed for a single target are unlikely to effectively treat these complex diseases. Network pharmacology is a novel method that combines system network analysis and pharmacology. Based on existing biological data, networks of target–disease, compound–compound, and compound–target interactions can be built to identify potential drug targets and clarify the action mechanism of a drug [17]. In recent years, target-network pharmacology (T-NP) has been introduced, which focuses on the relationship between the absorbable bioactive constituents and network pharmacology [18]. Compared with the conventional network pharmacology method, T-NP can reduce the false positive rate [18].

In this study, we aimed to: (1) isolate and identify lignans from *S. chinensis* using ultra high-performance liquid chromatography coupled with quadrupole time-of-flight mass spectrometry (UHPLC–Q-TOF-MS) in the plasma of dosed rats with AD; (2) clarify the mechanism of action of lignans from *S. chinensis* in treating AD (T-NP was used to integrate the absorbed constituents with the corresponding target proteins to generate a compound–target network); and (3) verify the related pathways and targets. To the best of our knowledge, this is the first study to reveal the possible metabolic pathways of lignans from *S. chinensis* in rats with AD and clarify the network pharmacology of their action mechanism in the treatment of AD.

## 2. Results

### 2.1. Identification of Absorbed Effective Constituents and their Metabolites from Lignans in S. chinensis

Pharmacokinetic studies on the main lignans from *S. chinensis* have proved that the peak time after oral administration ranges from 15 to 360 min [19]. The concentration–time curve of schisandrin, schisandrol B, and deoxyschizandrin from *S. chinensis* extract has showed the second peak at 480 min [20]. Based on these findings, we chose 30, 60, 120, 360, and 480 min as the time points to analyze the effective constituents absorbed in the blood. Using a blank plasma sample as the reference, we identified the characteristic ion peaks that only appeared in the plasma of dosed rats. The absorbed prototype constituents were then identified by comparing the peaks with the fragmentation information of the lignans, and some were also compared with those of the reference standards. The constituents identified are presented in Table S1. The following 10 lignans were found in the plasma of dosed rats as prototype constituents: schisandrin, gomisin D, benzoylgomisin H, angeloylgomisin Q, schisandrol B, gomisin G, gomisin K, gomisin E, deoxyschizandrin, and schisandrin B (Table 1). The prototype constituents absorbed into blood can be further metabolized by various drug-metabolizing enzymes in vivo. Drug metabolism is divided into two phase reactions, namely, phases I and II. In phase I reaction, the absorbed prototype constituents are usually oxidized or reduced. In phase II reaction, the constituents are mainly involved in binding reactions, and they conjugate to copolymers with small endogenous molecules, such as glucuronic acid, amino acids, and phosphate from phosphorylation [21]. A comparison of the characteristic ion peaks between the blank and dosed plasma samples revealed that there were some chromatographic ion peaks in only the plasma of dosed rats, in addition to the chromatographic ion peaks identified as prototype constituents. We speculated that these peaks were the metabolites of the prototype constituents. According to the principles of drug metabolism and related references [15], we extracted all the ion peaks that were consistent with possible metabolites of the prototype constituents absorbed into blood (within a mass error of 10 ppm). The collision energy was then adjusted to obtain fragment ions of the metabolites for the MS/MS analysis.

**Figure 1:**
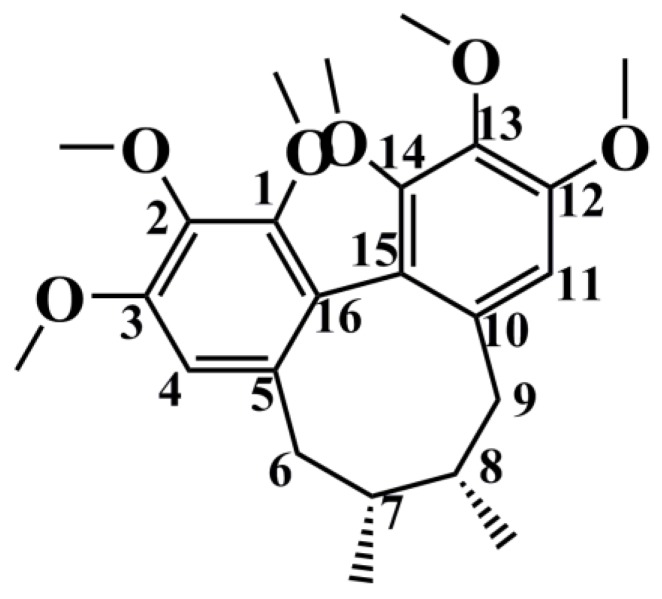
Chemical structure of deoxyschizandrin.

**Figure 2:**
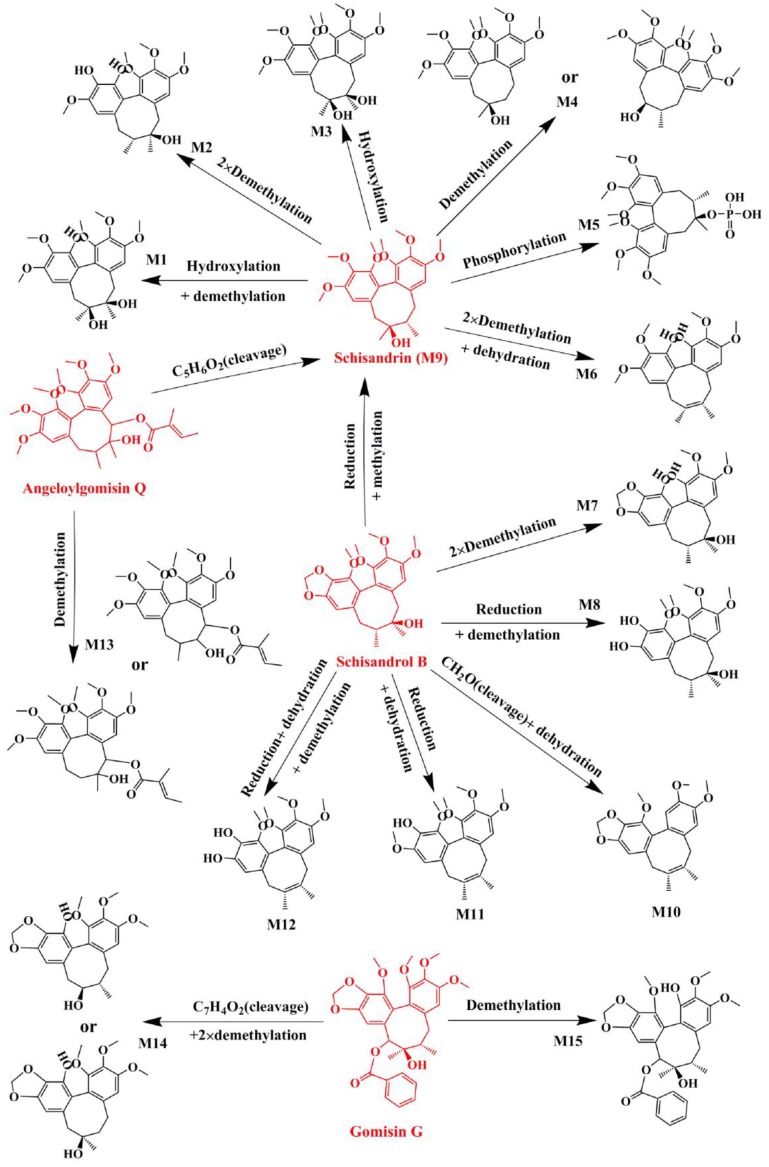
Proposed metabolic pathways of schisandrin, schisandrol B, angeloylgomisin Q, and gomisin G in the plasma of dosed rats with Alzheimer’s disease.

**Figure 3:**
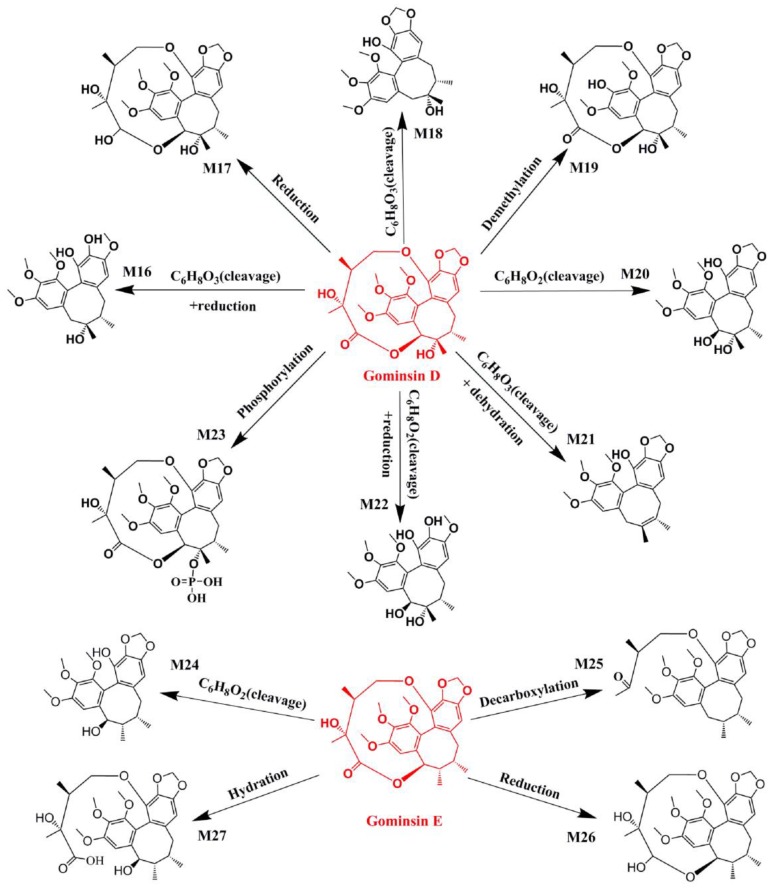
Proposed metabolic pathways of gomisin D and gomisin E in the plasma of dosed rats with Alzheimer’s disease.

**Figure 4:**
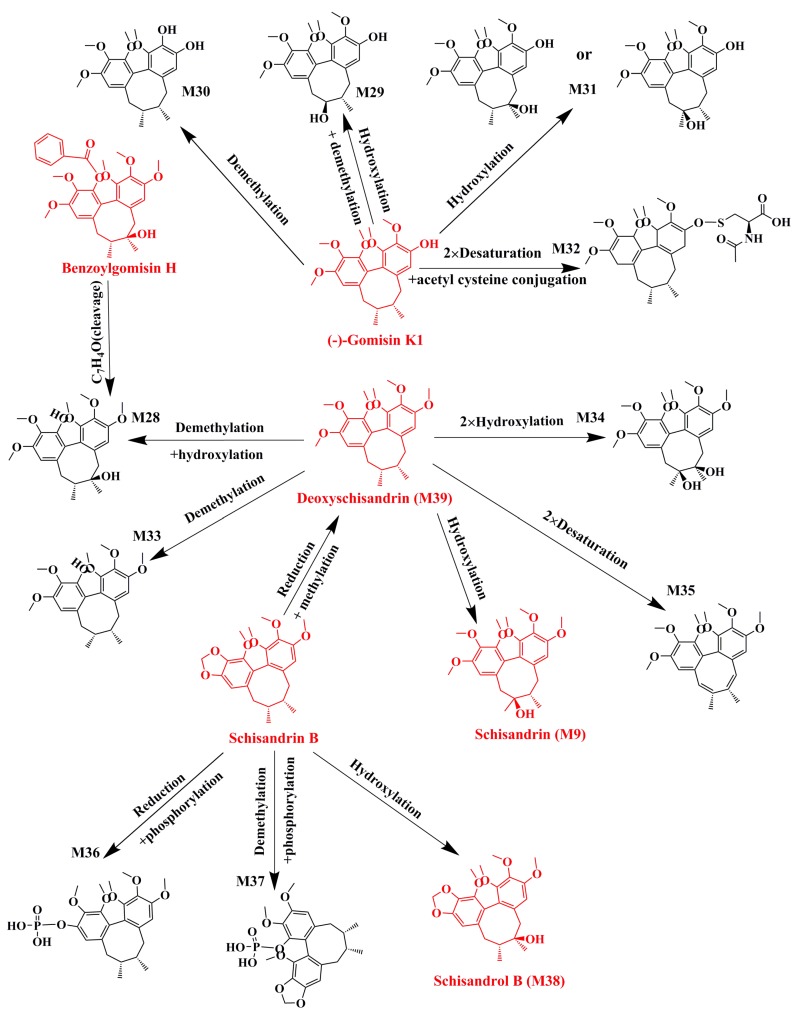
Proposed metabolic pathways of benzoylgomisin H, gomisin K, deoxyschizandrin, and schisandrin B in the plasma of dosed rats with Alzheimer’s disease.

For example, M9, M28, and M33-35 were identified as metabolites generated from deoxyschizandrin (Figure 1). M9 showed [M − H]^−^ ion at *m*/*z* 433.2210, which was 16 Da higher than that of the quasi-molecular ion of deoxyschizandrin. The mass change was similar to that of mono-oxidized deoxyschizandrin. M9 showed fragment ions at *m*/*z* 415, 400, 385, 384, 373, 369, 359, 354, 353, 338, and 322. M9 presented the same fragment ions with schisandrin. According to the MS/MS analysis results, M9 was identified as schisandrin. Studies have proved that deoxyschizandrin can be metabolized to schisandrin in rat [22]. M33 showed [M + H]^+^ ions at *m*/*z* 403.2093, which was 14 Da less than that of the prototype. The mass change corresponded to the loss of CH_2_ from deoxyschizandrin. M33 showed fragment ions at *m*/*z* 388, 385, 372, 370, 371, and 339. The characteristic fragment ions of M33 were consistent with those of schisanhenol [22,23]. M28 showed [M + H]^+^ ions at *m*/*z* 419.2061, which was 16 Da higher than that of M33. M28 showed fragment ions at *m*/*z* 401, 370, 369, and 337. The MS behavior of M28 was similar to that of M33. Therefore, demethylation and hydroxylation occurred at 1- and 7- or 8-positions, respectively. M34 showed [M + H]^+^ ions at m/z 449.2154, which was 16 Da higher than that of schisandrin. M34 showed fragment ions at *m*/*z* 431, 413, 398, and 383 by the successive loss of H_2_O, H_2_O, CH_3_, and CH_3_, respectively. The characteristic fragment ions of M34 were similar to those of schisandrin and deoxyschizandrin. Therefore, the hydroxylation occurred at 6-, 8-, and 9-positions, respectively. M35 showed [M + H]^+^ ions at *m*/*z* 413.1944, which was 36 Da higher than that of M34. M35 showed fragment ions at 398 and 383, which were consistent with the fragment ions 413 of M34. Therefore, desaturation mostly occurred at 6-, 7-, 8-, and 9-positions. Based on the above identification method, all possible metabolites of the prototype constituents absorbed into blood were identified. The MS and MS/MS data of the identified metabolites are summarized in Table 2. The main metabolic pathways of the lignans from *S. chinensis* included hydroxylation, demethylation, reduction, phosphorylation, and dehydration. The proposed metabolic pathways of the absorbed constituents are showed in Figure 2, Figure 3 and Figure 4.

### 2.2. Target Genes Related to the Identified Compounds

**Figure 5:**
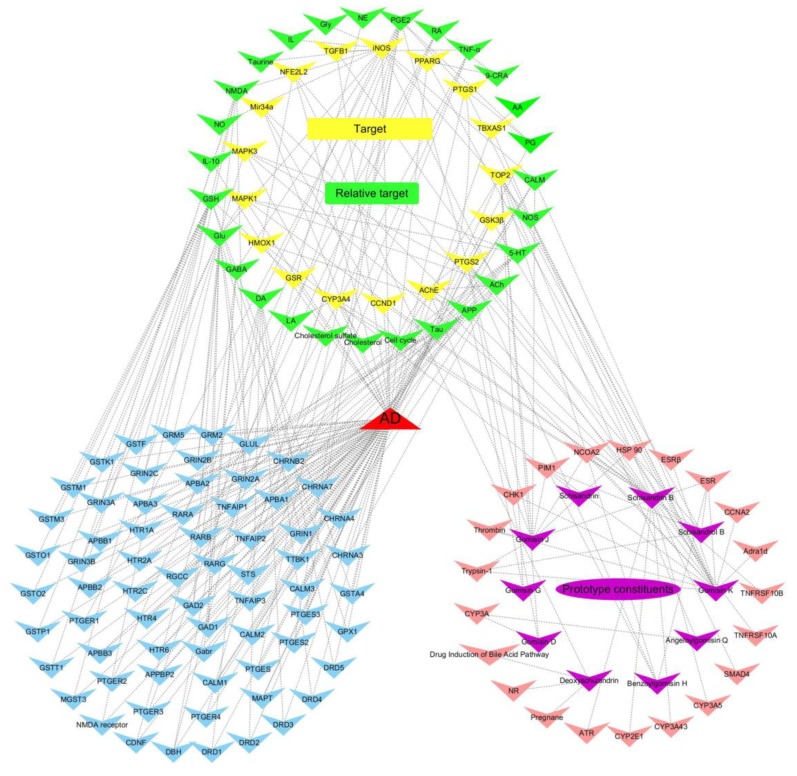
Absorbed effective constituent–target–disease network induced by the absorbed effective constituents of lignans from *S. chinensis*. The purple cycle indicates the absorbed effective constituents of lignans from *S. chinensis*, the yellow cycle is the common targets that contact the absorbed constituents and AD, the green cycle is the relative targets of AD and absorbed effective constituents, the blue cycle is the representative targets only related to AD, and the pink cycle is the targets only related to absorbed effective constituents; nodes represent for genes, proteins, endogenous compounds, and metabolic pathways.

To further illuminate the relationship between the absorbed effective constituents and disease pathways, an absorbed effective constituent–target–disease network was built. Using public databases (*viz.*, TCMSP, TCMID, TTD, Binding DB, OMIM, NCBI GENE), we identified approximately 2300 targets associated with AD and 38 targets in the absorbed effective constituents of lignans. Of all the targets, 17 target genes were shared by AD and absorbed effective constituents (Table 3), including acetyl cholinesterase (AChE), inducible nitric oxide synthase (iNOS), glycogen synthase kinase 3β (GSK3β), and heme oxygenase 1 (HMOX1). Those genes were utilized for further pathway analysis. The DAVID and KEGG databases were applied to enrich significantly correlated pathways (*P* < 0.05) that the targets were involved in. Meanwhile, the databases provided extended targets associated with these pathways. To obtain more potential therapeutic targets of lignans, a comparison was made between extended and AD-related targets. Hence, we obtained 27 targets that were closely related to lignans (Table 3). Finally, we input all the targets and constituents into Cytoscape and established the networks of effective constituents–targets–diseases (Figure 5).

### 2.3. Target Validation

According to the above potential target analysis, we found that the lignans from *S. chinensis* were associated with amyloid precursor protein (APP) metabolism, tau phosphorylation, neurotransmitter metabolism, nitric oxide (NO) anabolism, inflammatory response, retinol metabolism, lipid metabolism, glutathione (GSH) metabolism, oxidative stress, cell-cycle progression, and apoptosis. To clarify the mechanism of action of *S. chinensis* lignans in the treatment of AD, independent experimental validation based on the T-NP analysis results was performed.

#### 2.3.1. Effects of Lignans from *S. chinensis* on Aβ Deposition, *p*-tau Levels, and Number of Neurons in the Hippocampus of Rats with AD

**Figure 6:**
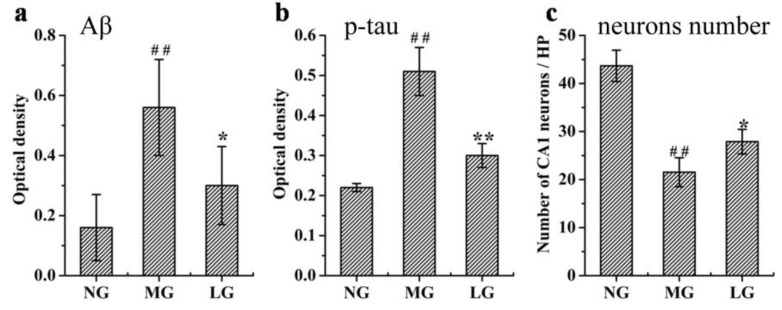
Effects of lignans from *S. chinensis* on the protein expression level of Aβ (**a**) and *p*-tau (**b**) and number of neurons (**c**) in the hippocampus of rats with AD. Notes: *n* = 10, per group; the data are expressed as mean ± SEM; compared with the normal control group (NG) by a *t*-test, ^##^ *P* < 0.01; compared with the AD model group (MG), ** *P* < 0.01, * *P* < 0.05.

The Aβ and *p*-tau levels, and hippocampal neuronal morphology affected by lignans form *S. chinensis* were analyzed (Figure 6). Compared with that of the normal control group (NG), the protein expression levels of Aβ and *p*-tau in the AD model group (MG) was significantly increased. Compared with that of MG, the lignin-treated group (LG) presented downregulation of Aβ and p-tau expression in the hippocampus of rats with AD. Compared with that of NG, the number of neurons in the hippocampus of MG was significantly decreased. Compared with that of MG, the number of neurons in the hippocampus of LG was increased.

**Figure 7:**
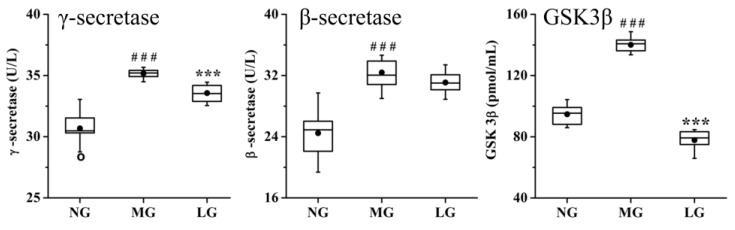
Effect of lignans from *S. chinensis* on the activity of γ-secretase, β-secretase, and GSK3β in the rat plasma. The box limits are in the 25th and 75th percentiles; the median is indicated by the horizontal bar; the whiskers are in the 1.5 interquartile ranges; the horizontal boundaries of the boxes represent the interquartile range and the black solid circle is the mean; data not included between the whiskers are plotted as an empty circle. Notes: n =10, per group; compared with NG by a *t*-test, ^###^ *P* < 0.001; compared with MG, *** *P* < 0.001.

The activity of enzymes involved in the production of Aβ and p-tau was analyzed (shown in Figure 7). Compared with that in NG, the activity of γ-secretase, β-secretase, and GSK-3β in MG was significantly increased. Compared with that in MG, the activity of γ-secretase and GSK-3β in LG was decreased, but the lignans from *S. chinensis* could not downregulate the activity of β-secretase.

#### 2.3.2. Effects of Lignans from S. chinensis on Inflammatory and Oxidant Damage in Rats with AD

**Figure 8:**
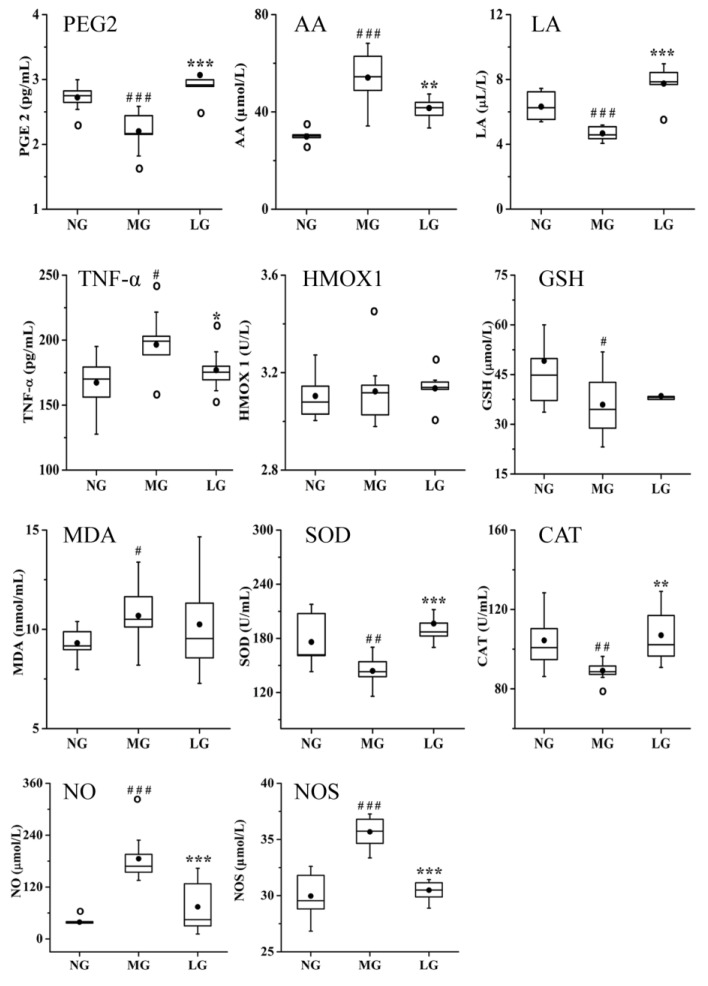
Effect of lignans from *S. chinensis* on the levels of prostaglandin E2 (PGE2), arachidonic acid (AA), linoleic acid (LA), tumor necrosis factor-α (TNF-α), heme oxygenase 1 (HMOX1), glutathione (GSH), malondialdehyde (MDA), superoxide dismutase (SOD), and catalase (CAT), nitric oxide (NO), and nitric oxide synthase (NOS) in the rat plasma. The box limits are in the 25th and 75th percentiles; the median is indicated by the horizontal bar; the whiskers are in the 1.5 interquartile ranges; the horizontal boundaries of the boxes represent the interquartile range and the black solid circle is the mean; data not included between the whiskers are plotted as an empty circle. Notes: *n* = 10, per group; compared with NG by a *t*-test, ^###^ *P* < 0.001, ^##^ *P* < 0.01, ^#^ *P* < 0.05; compared with MG, *** *P* < 0.001, ** *P* < 0.01, * *P* < 0.05.

Studies have shown that inflammation plays an important role in the pathogenesis of AD. Oxidative stress reaction can stimulate inflammation and Aβ production, resulting in AD aggravation [24]. Based on the results of the T-NP analysis, the related biochemical parameters involved in inflammatory and oxidant damage were determined, and the results are shown in Figure 8. Compared with that in NG, the levels of arachidonic acid (AA), tumor necrosis factor-α (TNF-α), malondialdehyde (MDA), NO, and nitric oxide synthase (NOS) was significantly increased, whereas the level of prostaglandin E2 (PGE2), linoleic acid (LA), GSH, superoxide dismutase (SOD), and catalase (CAT) were significantly decreased in MG. The level of HMOX1 was not significantly different between NG and MG. Compared with that in MG, the levels of PGE2, LA, SOD, and CAT were significantly increased, whereas the levels of TNF-α, NO, NOS, and AA were significantly decreased in LG.

#### 2.3.3. Effects of Lignans from S. chinensis on Neurotransmitters in Rats with AD

**Figure 9:**
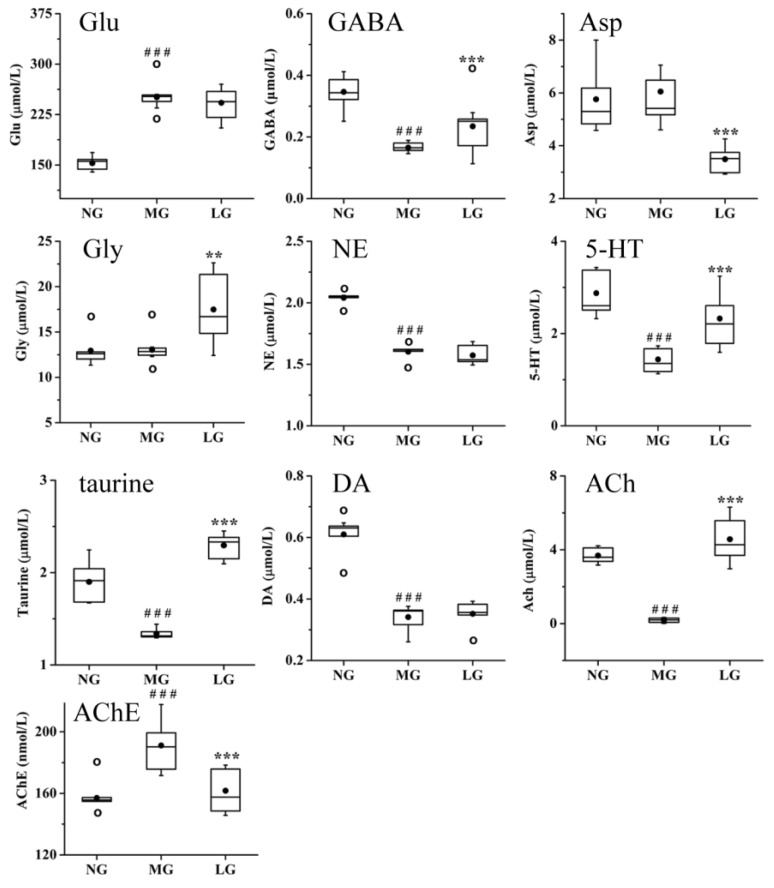
Effect of lignans from *S. chinensis* on the level of Glu, γ-aminobutyric acid (GABA), aspartic acid (Asp), glycine (Gly), norepinephrine (NE), 5-hydroxytryptamine (5-HT), taurine, dopamine (DA), acetylcholine (ACh), and acetyl cholinesterase (AChE) in the plasma of rats with AD. The box limits are in the 25th and 75th percentiles; the median is indicated by the horizontal bar; the whiskers are in the 1.5 interquartile ranges; the horizontal boundaries of the boxes represent the interquartile range and the black solid circle is the mean; data not included between the whiskers are plotted as an empty circle. Notes: n = 10, per group; compared with NG by a *t*-test, ^###^ *P* < 0.001; compared with MG, *** *P* < 0.001, ** *P* < 0.01.

Neurotransmitters participate in the regulation of various physiological functions, such as emotions, sleep, learning, and memory, which have a close relationship with the pathogenesis of AD [25,26,27,28]. Based on the results of the T-NP analysis, 10 neurotransmitters and 2 enzymes were determined to validate whether they were potential targets of lignans from *S. chinensis* in the treatment of AD. The results are shown in Figure 9. Compared with those in NG, the levels of glutamic acid (Glu) and AChE were significantly increased, but the levels of γ-aminobutyric acid (GABA), norepinephrine (NE), 5-hydroxytryptamine (5-HT), taurine, dopamine (DA), and ACh were significantly decreased in MG. The lignans from *S. chinensis* can upregulate the level of GABA, 5-HT, ACh, and taurine and downregulate the level of AChE in rats with AD. Although there was no significant difference in the levels of aspartic acid (Asp) and glycine (Gly) between NG and MG, compared with those in MG, the level of Asp was significantly decreased and the level of Gly was significantly increased in LG. Lignans from *S. chinensis* had no significant regulation on the level of NE, DA, and Glu.

#### 2.3.4. Effects of Lignans from S. chinensis on other Pathways in Rats with AD

**Figure 10:**
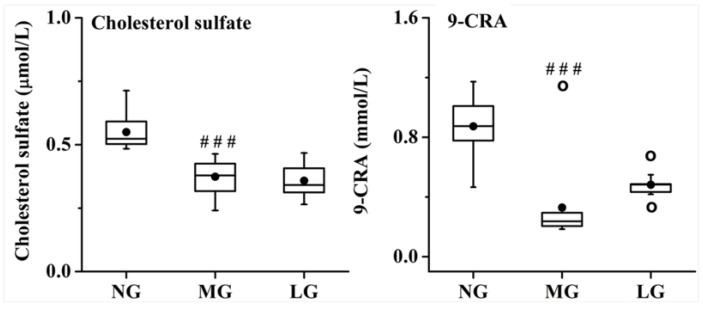
Effect of lignans from *S. chinensis* on the level of cholesterol sulfate and 9-*cis*-retinoic acid (9-CRA) in the plasma of rats with AD. The box limits are in the 25th and 75th percentiles; the median is indicated by the horizontal bar; the whiskers are in the 1.5 interquartile ranges; the horizontal boundaries of the boxes represent the interquartile range and the black solid circle is the mean; data not included between the whiskers are plotted as an empty circle. Notes: *n* = 10, per group; compared with NG, ^###^ *P* < 0.001.

Based on the results of the T-NP analysis, the level of cholesterol sulfate and 9-*cis*-retinoic acid (9-CRA) was determined to validate whether they were potential targets of lignans from *S. chinensis* in the treatment of AD. The results are shown in Figure 10. Compared with that in NG, the level of cholesterol sulfate and 9-CRA was significantly decreased in MG. However, the lignans from *S. chinensis* had no significant regulation on the level of cholesterol sulfate and 9-CRA.

## 3. Discussion

### 3.1. Effects of Lignans from S. chinensis on the Aβ and p-tau Levels, and Hippocampal Neuronal Morphology in Rats with AD

Pathologically, AD involves neurofibrillary tangles (NFTs), Aβ deposition, and neuronal loss. Glycogen synthase kinase-3 (GSK-3) is a serine/threonine kinase that is activated by tubulin and is involved in the formation of paired helical filaments. Tau is the main component of NFTs in AD. GSK3β phosphorylates tau in a site-specific manner so that tau binds and stabilizes the microtubule structure [29]. The activity of GSK-3 contributes to both Aβ production and neuronal death mediated by Aβ [30]. Gomisin K interacts with GSK3β, indicating that lignans from *S. chinensis* might affect NFTs, Aβ deposition, and neuronal loss. DNA topoisomerase controls and alters the topologic states of DNA during transcription. DNA topoisomerase II Alpha (Top2α) and DNA topoisomerase 2-beta (Top2β) are two subtypes of Top2. Further, Top2α mainly regulates cell multiplication and development [31,32], while Top2β is mainly involved in neuronal cells survival, development, and differentiation [32,33,34,35]. MicroRNA 34a (mir34a) is a member of the highly conserved mir-34 family. It has been reported that the miRNA pathways dramatically modulate polyglutamine and tau-induced neurodegeneration [36]. Mir34a is related to cell-cycle progression and apoptosis [37]. The loss of mir34 triggers a genetic profile of accelerated brain aging, late-onset brain degeneration, and a catastrophic decline in survival, whereas the upregulation of mir34 extends median life span and mitigates neurodegeneration induced by human pathogenic polyglutamine disease protein [38]. Cyclin D1 (CCND1) belongs to the highly conserved cyclin family, whose members play important roles throughout the cell cycle. Cyclins are regulators of cyclin-dependent kinases (CDKs). Recently, studies have indicated that the deregulation of the neuronal cell cycle plays an important role in several central nervous system diseases [39,40], and is also a significant contributor to regionally specific neuronal death in AD [41]. The interaction between the above targets and *S. chinensis* lignans’ active constituents, including gomisin K, angeloylgomisin Q, benzoylgomisin H, schisandrol B, gomisin D, gomisin G, and schisandrin B, suggests that the lignans from *S. chinensis* might regulate neuronal cell-cycle progression and apoptosis in rats with AD. Additionally, according to the results of the T-NP analysis, it was found that NFTs, Aβ deposition, or neuronal loss were also closely related to other targets affected by lignans, such as AChE and HMOX1. This observation indicates that NFTs, Aβ deposition, or neuronal loss might play an important role in the therapeutic effect of lignans from *S. chinensis* in AD treatment. The β- and γ-secretases take part in releasing Aβ, which has neurotoxic effect [42,43]. β-Secretase is a rate-limiting enzyme in APP degradation and γ-secretase is a rate-limiting enzyme in Aβ production [44]. The lignans from *S. chinensis* can alleviate Aβ deposition, tau hyperphosphorylation, and neuronal dysfunction and death in the brain of rat with AD. γ-Secretase and GSK-3β were considered potential targets of *S. chinensis* lignans for regulating Aβ deposition and hyperphosphorylated forms of tau. These results indicate that lignans from *S. chinensis* could affect the activity of GSK-3β in rats with AD, thus verifying the correctness and reliability of the T-NP analysis. By verifying the relevant targets, we found that Aβ, γ-secretase, tau, and neuronal dysfunction and death are effective therapeutic targets of the lignans from *S. chinensis* for treating AD.

### 3.2. Effects of Lignans from S. chinensis on Inflammatory and Oxidant Damage in Rats with AD

Inflammation and oxidative oxidation might stimulate the production of Aβ, resulting in the aggravation of AD. Neuronal nitric oxide synthase (nNOS), along with iNOS and endothelial nitric oxide synthase (eNOS), synthesizes NO from L-arginine and molecular oxygen [45,46]. Nitric oxide is a reactive free radical and shares several properties with a neurotransmitter in the nervous system. It has multiple cellular molecular targets, including N-methyl-D-aspartate, Glu, ACh, DA, NE, GABA, taurine, and Gly [47]. Furthermore, NO is involved in inflammation and enhances the synthesis of proinflammatory mediators such as interleukin-6 (IL-6) and interleukin-8 (IL-8) [48]. Prostaglandin-endoperoxide synthase (PTGS) is a key enzyme in prostaglandin (PG) biosynthesis and is divided into a constitutive PTGS1 and an inducible PTGS2; it catalyzes the conversion of AA to PG [49]. PTGS2 is responsible for the production of inflammatory PGs. Both iNOS and PTGS2 are major inflammatory mediators [50]. Arachidonic acid is an essential polyunsaturated fatty acid in organisms that can mediate inflammation [51]. In organisms, LA can be converted to AA. The cytochrome P450 proteins are monooxygenases that catalyze various reactions, including metabolism of drugs and synthesis of cholesterol, steroids, and other lipids [52,53,54]. Thromboxane A synthase 1 (TBXAS1) is a member of the cytochrome P450 superfamily of enzymes. However, TBXAS1 is considered a member of the cytochrome P450 superfamily based on the sequence similarity rather than functional similarity [55]. TBXAS1 is associated with bleeding disorders. The cytochrome P450 family 3 subfamily A member 4 (CYP3A4) is an important paralog of TBXAS1. In organisms, CYP3A4 participates in the conversion of LA to epoxyoctadecenoic acid, which is the peroxidation product of LA. It has been reported that treatment with schisandrin B and schisandrin A for three days can inhibit the activity of CYP3A4 and increase the concentration and oral bioavailability of drug metabolized by CYP3A [56,57]. Li et al. investigated the inhibitory effects of schisandrin A and schisandrin B on CYP3A activity in rat liver microsomes and found that schisandrin-induced inhibition is mostly attributable to a mixed noncompetitive and complete inhibition [58]. Transforming growth factor β1 (TGFB1), mitogen-activated protein kinase 1 (MAPK 1), and MAPK3 belong to the MAPK signaling pathway. These three targets are associated with cellular responses to proinflammatory cytokines [59]. Both the TNF and MAPK signaling pathways are closely related to the damage induced by cytotoxic drugs, irradiation, heat shock, reactive oxygen species, and lipopolysaccharide. Studies have shown that schisandrol B can suppress the NF-κB and MAPK signaling pathways to inhibit lipopolysaccharide-induced inflammatory responses in microglia cells [60]. Additionally, MAPKs can regulate cell survival and apoptosis [61]. The MAPK signaling pathway affects the glutamatergic, cholinergic, and serotonergic synapse pathways. Extracellular signal-regulated kinase (ERK) is a member of the MAPK family. Studies have found that ERK is involved in synaptic plasticity, learning, and memory in the brain [62]^.^ Gomisin K, benzoylgomisin H, schisandrol B, schisandrin B, and gomisin D were found to interact with iNOS, PTGS1, PTGS2, TGFB1, and MAPK, indicating that these compounds exerted anti-inflammatory effects. In the present study, we speculated that the anti-inflammatory effects might be one of the ways by which the lignans from *S. chinensis* improve learning and memory in rats with AD. The results of target validation showed that the lignans in *S. chinensis* alleviated inflammation in rats with AD by directly regulating the level of TNF-α, PGE2, NO, and NOS, and these results are consistent with those of the T-NP analysis. The lignans from *S. chinensis* alleviated the abnormal metabolism of AA and LA. The inhibitory effects of lignans from *S. chinensis* on the activity of CYP3A4 might inhibit the peroxidation of LA, thus increasing the level of LA in rats with AD. Lignans from *S. chinensis* might promote the metabolism of AA to PGs and inhibit the metabolism of LA to AA, thus increasing the anti-inflammatory ability in organisms.

Tumor necrosis factor-α is a pro-inflammatory cytokine and PGE2 is a member of PG with immunosuppressive and anti-inflammatory effects. The results of the present study showed that the lignans from *S. chinensis* alleviated inflammation and oxidative stress in rats with AD by directly regulating the level of TNF-α, PGE2, SOD, and CAT.

In addition to NOS, other targets such as GSH and HMOX were found to be associated with the antioxidant activity of lignans from *S. chinensis*. Glutathione-disulfide reductase (GSR) is a central enzyme of cellular antioxidant defense, and it reduces oxidized glutathione disulfide (GSSG) to the sulfhydryl form, GSH, which is an important cellular antioxidant [63]. The liver is considered the central organ of GSH metabolism, and the concentration of GSH in plasma and erythrocytes could reflect the ability of the liver to synthesize GSH. Nuclear factor erythroid 2-related factor 2 (NFE2L2) is important for the coordinated up-regulation of genes in response to oxidative stress. It negatively regulates the oxidative stress-induced intrinsic apoptotic signaling pathway [64]. The *HMOX1* gene encodes the HMOX protein, which has antioxidative, neuroprotective, and potentially anti-inflammatory effects [65,66]. The binding of APP inhibits the HMOX activity, and the absence of HMOX activity might contribute to neuronal cell death in patients with AD [66]. The active constituents of lignans from *S. chinensis,* including schisandrin B, schisandrin, and deoxyschizandrin, might regulate the above targets, indicating that the lignans might have antioxidant and anti-inflammatory effects. Studies have shown that schisandrin B acts as an antioxidant to prevent cerebral oxidative stress and tert-butylhydroperoxide-induced cerebral toxicity in mice [67]. Malondialdehyde is a product of lipid peroxidation. The level of MDA in the plasma indirectly reflects the severity of free radical attack on body cells. Glutathione, CAT, HMOX1, and SOD are all important antioxidants in organisms, and they have several important physiological functions, such as scavenging free radicals, and maintaining normal cell growth and cellular immunity [63,65,66,68,69]. Therefore, the levels of these compounds constitute an important standard by which the antioxidant ability of an organism can be evaluated. In the present study, the lignans from *S. chinensis* alleviated oxidative stress in rats with AD by directly upregulating the level of SOD and CAT.

### 3.3. Effects of Lignans from S. chinensis on Neurotransmitters in Rats with AD 

As an important part of the nervous system, neurotransmitters play the role of chemical messengers in the transmission of nerve signals. They are closely related to the learning and memory of an organism. In recent years, the close relationship between neurotransmitters and AD pathogenesis has attracted attention [70,71].

Cholinergic neurons in the hippocampus are crucially involved in learning and memory, and their dysfunction can lead to a severe form of dementia [72]. Acetylcholine is a key neurotransmitter of the cholinergic system. As a molecular chaperone, AChE is involved in Aβ aggregation and deposition in the pathogenesis of AD. Both the peripheral anionic site and the N-terminal region of AChE have been indicated as Aβ binding domains [73]. Acetylcholine esterase aggregates in the nucleus of neuroblastoma SK-N-SH cells can promote the process of neuroblastoma cell apoptosis [74]. Programmed cell death plays an integral role in AD. The active constituents of lignans from *S. chinensis*, including gomisin K, might affect Aβ accumulation and neuronal apoptosis by acting on AChE. Lignans from *S. chinensis* could decrease the level of AChE to alleviate the lack of ACh, affecting Aβ deposition and neuronal apoptosis.

5-Hydroxytryptamine belongs to the serotonergic systems, which participate in the regulation of various physiological functions, such as emotions, sleep, appetite, learning, and memory. The reduction in 5-HT level in the brain impairs the memory of patients with AD [75]. In the present study, we found that the lignans from *S. chinensis* could increase the level of 5-HT in rats with AD, indicating that these lignans might regulate the dysfunction of serotonergic systems in rats with AD.

Amino acid neurotransmitters contain Asp, Glu, Gly, and GABA. Gly and GABA exert postsynaptic inhibition and neuroprotective effects [76]. Taurine has neuroprotective effects in organisms, whereas Asp has neurotoxic effects [25]. The active constituents of lignans from *S. chinensis* exert their neuroprotective effects by decreasing the level of Asp and increasing the level of taurine, Gly, and GABA. These results indicate that the lignans from *S. chinensis* alleviate the dysfunctions in amino acid neurotransmitter, GABAergic, serotonergic, and cholinergic systems in rats with AD. Combining these findings with the results on the antioxidants and neurotransmitters, we found that the lignans from *S. chinensis* mainly upregulated the level of enzymes that are protective against oxidative stress and neuroprotective amino acid neurotransmitters. We speculated that the lignans from *S. chinensis* have a significant role in the protection of the central nervous system during the pathological process of AD, and this might be the main reason for its ability to treat AD.

### 3.4. Effects of Lignans from S. chinensis on other Pathways in Rats with AD

Peroxisome proliferator-activated receptor gamma (PPARG) is a regulator of adipocyte differentiation [77] and glucose homeostasis. Peroxisome proliferator activated receptors (PPARs) form heterodimers with retinoid X receptors (RXRs), and these heterodimers regulate the transcription of various genes [78]. Additionally, PPARG has been implicated in obesity, diabetes, and atherosclerosis. Some evidence demonstrates that chronic over-nutrition induces metabolic stress and neuroinflammation in the brain. [79]. Nutraceuticals might represent the future of preventing and tackling neurodegenerative disorders. The active constituent of lignans from *S. chinensis*—gomisin K—interacts with PPARG, indicating that these compounds might participate in nutrient metabolism. However, the results of target validation showed that the lignans from *S. chinensis* did not significantly regulate cholesterol sulfate and 9-CRA.

## 4. Materials and Methods

### 4.1. Chemicals and Materials

Aβ25–35 and cholesterol sulfate were obtained from Sigma-Aldrich (St. Louis, MO, USA). AA, 9-CRA, Glu Asp, GABA, Gly, Ach, 5-HT, DA, and NE were purchased from Aladdin (Shanghai, China). LA was purchased from Fluka (Muskegon, MI, USA). Leucine enkephalin and sodium formate were obtained from Waters (Milford, MA, USA). Acetonitrile, methyl alcohol, and formic acid (HPLC-grade) were obtained from Tedia Company, INC. (Cincinnati, OH, USA). Ethanol absolute was purchased from Beijing Chemical (Beijing, China). Ethyl acetate was purchased from Tian in Fuyu Fine Chemical Co., Ltd (Tianjing, China). Ultrapure water was obtained using Milli-Q water purification system (Milford, MA, USA). *Schisandra chinensis* was purchased from Kaida Company (Baoding, Hebei, China), and authenticated by researcher Fengrui Song, Center for Mass Spectrometry in Changchun. AB-8 macroreticular adsorption resin was purchased from Amicogen Biopharm Co. Ltd (Jining, Shandong, China). The assay kits for β-secretase, γ-secretase, GSK-3β, AChE, TNF-α, PGE2, GSH, HMOX1, SOD, NOS, NO, MDA, and CAT were obtained from Nanjing Jiancheng Bioengineering Institute (Nanjing, Jiangsu, China).

### 4.2. Establishment of the AD Model and Drug Administration

Male Sprague–Dawley rats (weights of 200 ± 20 g) were provided by the Experimental Animal Center of Jilin University (Changchun, Jilin, China). They were maintained in accordance with the guidelines for the care and use of laboratory animals. All rats were maintained in a barrier system with regulated temperature (21 ± 2 °C) and humidity (50 ± 5%) under a light–dark cycle of 12 h per day. All the rats were acclimated for one week before experimentation. Aβ25–35 was used to establish the AD rat model. A small animal stereotaxic frame was purchased from All Points Industrial Supply (Westminster, MA, USA). The rat model was established as previously described [80]. Briefly, the rats were anesthetized with pentobarbital sodium, and then placed on a stereotaxic apparatus. After cleaning the rats’ scalp with iodine solution and incising at the midline to expose the skull, we injected Aβ25–35 solution into the bilateral hippocampus. The rats were then randomly divided into two groups, and each group contained 10 rats. One group was selected as the AD model control group, and the rats were administered saline. Whereas the rats from the other group were orally administered purified lignans isolated from *S. chinensis* at a dose of 0.55 g crude drugs per kilogram per day. To analyze the effective constituents absorbed into blood, the rats were treated with extracts of lignans isolated from *S. chinensis*. Oral administration was performed once per day for eight days. All animal studies were performed according to the Institutional Guidelines for the Care and Use of Laboratory Animals, and the study protocols were approved by the Animal Research Ethics Committee of Jilin University.

### 4.3. Effective Constituents Absorbed in the Blood

#### 4.3.1. Plasma Sample Collection and Preparation

After the last administration of extract, blood samples were collected from the oculi chorioideae vein into tubes containing heparin sodium at 30, 60, 120, 360, and 480 min. The amount of blood withdrawn was 300 μL at each time point. The collected blood samples were centrifuged at 7000 rpm for 15 min at 4 °C to obtain the plasma. Then, 100 μL of plasma was mixed with 400 μL of cold methyl alcohol. The mixture was vortexed for 30 min, and then centrifuged at 13 000 rpm for 15 min at 4 °C to collect the supernatant, which was dried under N_2_. Thereafter, the plasma samples were redissolved with 20 μL of methyl alcohol, vortexed for 2 min, and then recentrifuged. The supernatants were used for the UHPLC–Q-TOF-MS analysis.

#### 4.3.2. UHPLC–MS Conditions and Data Processing of Plasma Analysis

The chromatographic analysis was performed using Waters Acquity Ultra-Performance Liquid Chromatography system coupled with a Q-TOF SYNAPT G2 High Definition Mass Spectrometer (Waters, Manchester, UK). The chromatographic separation was carried out on a Waters Acquity UPLC BEH C18 column (2.1 mm × 50 mm, 1.7 μm). The column temperature was maintained at 40 °C and the injection volume was 5 μL. The mobile phase consisted of 0.1% formic acid (*v*/*v*) (A) and acetonitrile (B) at a flow rate of 0.35 mL/min. The gradient elution in positive mode was performed as follows: 5–50% B at 0–1 min, 50–51.5% B at 1–5 min, 51.5–52.5% B at 5–6 min, 52.5–54% B at 6–11 min, 54–70% B at 11–11.5 min, 70–100% B at 11.5–15 min, 100–5% B at 15–15.5 min, and 5% B at 15.5–20 min. The sample manager temperature was set at 4 °C. An electrospray ionization (ESI) source with a scanning mass-to-charge (*m*/*z*) range from 50 to 1000 Da was used. The source temperature was 120 °C. Nitrogen was used as cone and desolvation gas. The flow rates were 50 and 700 L/h, respectively, and the solvent temperature was 350 °C. The cone and extraction cone voltages were 40 and 5.0 V, respectively. The capillary voltage was set at 3.0 kV. The quality standard curves were established using sodium formate. Leucine enkephalin was used as the reference mass. The MS^E^ data acquisition was performed using He as collision gas with a low collision energy of 5 eV and a high collision energy of 10–60 eV. The raw data were obtained using UNIFI™. The database of lignans in *S. chinensis* based on the MS databases (PubMed, Mass Bank, Chemspider, and METLIN) and references [16,81,82,83,84,85] was built using UNIFI™ (Waters Corp.). The database of lignans in *S. chinensis* included compound name, molecular formula, structural formula, and fragment ion. Then, MassFragment™ and UNIFI™ were used to analyze raw data of purified lignans isolated from *S. chinensis* [21]. The results are shown in Supporting Information Figure S1. Using the blank plasma sample as the reference, based on the results of identification of the purified lignans isolated from *S. chinensis*, the prototype constituents in the plasma of dosed rats with AD were selected. Their metabolic pathways were then identified individually from expected metabolic pathways including demethylation, hydroxylation, desaturation, hydroxylation, dehydration, reduction, methylation, and phosphorylation (Table 4).

### 4.4. Network Construction and Pathway Analyses

Information about interactions between the absorbed effective constituents of lignans from *S. chinensis* and AD is widely dispersed among numerous databases and published studies. Thus, we collected useful information from the related databases. Firstly, we input the 10 prototype compounds individually into these databases, including TCMSP (version 2.3, http://sm.nwsuaf.edu.cn/lsp/tcmsp.php), STITCH (version 5.0, http://stitch1.embl.de), TCMID (version 2.0, http://www.megabionet.org/tcmid/), TTD (http://bidd.nus.edu.sg/group/TTD/ttd.asp), and BindingDB (https://www.bindingdb.org/bind/index.jsp), to identify the targets associated with the absorbed effective constituents in *Homo sapiens*. Secondly, the targets involved in AD were retrieved utilizing the OMIM (http://omim.org/about), NCBI GENE (http://www.ncbi.nlm.nih.gov/gene), TCMID, TCMSP, and TTD. Thirdly, the identified molecular targets were utilized for further pathway analysis using DAVID (version 6.8, http://david.niaid.nih.gov) and KEGG (https://www.kegg.jp/kegg/kegg1.html). All targets related to lignans’ effects on AD, including genes, proteins, endogenous compounds and metabolic pathways, were selected. The principal methods for searching the relevant databases were applied according to the available literature [86,87,88,89,90,91,92]. Cytoscape 3.6.1 (http://www.cytoscape.org/) is an open-source software tool that enables users to analyze, visualize, and publish complex networks, primarily for a vast amount of biological data [93]. Finally, we input the data collected by the first three steps into Cytoscape and established the networks of effective constituents–targets–diseases.

### 4.5. Target Validation

Thirty rats were randomly divided into the following three groups: the normal control group (NG), AD model group (MG), and lignin-treated group (LG). The AD model was established according to the method described in 2.4. The MG and LG rats were administered Aβ 25–35 solution via injection into the bilateral hippocampus, while the NG rats were administered sterile physiological saline via injection into the bilateral hippocampus. Then, according to the dose of the drug in 2.2, the LG rats were treated with purified lignans isolated from *S. chinensis* for two months. After treatment for the specified duration, all of the rats were sacrificed.

The hippocampus samples were stored in formalin solution to be stained with hematoxylin and eosin (H&E) to observe histopathological changes and immunohistochemical staining to observe the protein expression of Aβ and p-tau in the hippocampus. The detailed measurement methods are based on a previous study [79].

Whole blood samples from the rats were collected and centrifuged at 3 000 rpm for 10 min at 4 °C to obtain plasma samples. The level of TNF-α, PGE2, GSH, HMOX1, SOD, NOS, NO, MDA, and CAT was measured in plasma using an enzyme-linked immunosorbent assay (ELISA) kit.

For the quantitative analysis of neurotransmitters, 200 μL of plasma was mixed with 800 μL of cold acetonitrile or 50% acetonitrile–water, vortexed for 1 min, left standing for 10 min and finally centrifuged at 13 000 rpm for 15 min at 4 °C. The supernatants were analyzed quantitatively by UHPLC–MS/MS.

The chromatographic analysis was performed using Waters Acquity Ultra-Performance LC system (Waters) coupled with a Xevo TQ MS spectrometer with an ESI source (Waters, Manchester, UK). For quantitation of neurotransmitters in plasma, the chromatographic separation was performed using the Venusil ASB C18 HPLC column (4.6 mm × 250 mm, 5 mm) from Agela Technologies. The injection volume was 20 μL. The mobile phase consisted of 0.1% formic acid-water (A) and 33% methyl alcohol–water (containing 0.06% formic acid) (B) at a flow rate of 0.5 mL/min. Gradient elution in the positive mode was performed as follows: 0% B at 0–3.5 min, 0–22% B at 3.5–4 min, 22–24% B at 4–8 min, 24–100% B at 8–8.5 min, 100% B at 8.5–15.5 min, 100–0% B at 15.5–16 min. For the quantitative analysis of AA, 9-CRA, cholesterol sulfate, and LA in plasma, the chromatographic separation was carried out on a Waters Acquity UPLC BEH C18 column (2.1 mm × 50 mm, 1.7 μm). Their respective cone voltages and collision energies are listed in Table 5. The injection volume was 10 μL. The mobile phase consisted of 0.1% formic acid (*v*/*v*) (A) and acetonitrile (B) at a flow rate of 0.35 mL/min. The gradient elution was performed as follows: 5–51% B at 0–1 min, 51–63.5% B at 1–6 min, 63.5–82% B at 6–7 min, 82–83.5% B at 7–9 min, 83.5–100% B at 9–12 min, 100–5% B at 12–12.1 min, 5% B at 12.1–17 min. The column temperature and sample manager temperature were respectively maintained at 40 and 4 °C.

The MS analysis was operated in the positive and negative modes of a triple quadrupole mass spectrometer. The source temperature was 150 °C. The solvent temperature was 350 °C. The other optimized parameters are showed in Table 5. Argon was used as the collision gas and nitrogen was used as nebulizer gas and desolvation gas. The total ion current chromatograms were obtained using a mass spectrometer in multiple monitoring modes.

## 5. Conclusions

In this study, the absorbed constituents and metabolites of lignans isolated from *S. chinensis* in the plasma of rats with AD were separated and identified by UHPLC–Q-TOF-MS. Eleven absorbed prototype constituents and 39 metabolites were identified or tentatively characterized in the plasma of dosed rats. The major metabolic reactions of the absorbed prototype constituents were determined to be hydroxylation, demethylation, reduction, phosphorylation, and dehydration. Based on the absorbed prototype constituents in the blood, a new T-NP approach was used to investigate the pharmacodynamic material basis and the pharmacologic mechanism of lignans in *S. chinensis* for the treatment of AD. Seventeen target genes involved in AD were found to be associated with multiple active pharmaceutical ingredients of lignans from *S. chinensis,* including AChE, iNOS, GSK3β, and HMOX1. These target genes were also associated with other targets including APP, neurotransmitters, and inflammatory response molecules, all of which are involved in AD. To further reveal the pharmacologic mechanism of lignans from *S. chinensis* in alleviating the damage to memory and cognitive function in rats with AD, independent experimental validation based on T-NP was performed. It was found that the lignans from *S. chinensis* regulated several pathways in rats with AD, including APP metabolism, NFTs, neurotransmitter metabolism, inflammatory response, and the antioxidant system. Overall, the results provide valuable information and support for further studies on the pharmacology and in vivo action mechanism of lignans from *S. chinensis.* The combination of T-NP and independent experimental validation could be used as a simple, quick, and feasible approach to decipher the chemical and pharmacological bases of other natural products. This study could help researchers elucidate the underlying multi-compound, multi-target, and multi-pathway modes of actions of herbal medicines. It also offers a novel research strategy for studying the action mechanisms of other herbal medicines.

## Figures and Tables

**Table 1:** Absorbed prototype constituents in dosed Alzheimer’s disease (AD) rat plasma.

No.	Parent Compound	Rt (min)	Measured Mass	Formula	Time Points (min)
P1	Schisandrin ^a^	2.17	433.2210	C_24_H_32_O_7_	30, 60, 120
P2	Gomisin D	2.37	553.2228	C_28_H_34_O_10_	30, 60, 120, 360, 480
P3	Schisandrol B ^a^	2.55	439.1723	C_23_H_28_O_7_	30, 60, 120
P4	Benzoylgomisin H	3.12	523.2281	C_30_H_34_O_8_	60, 120, 360
P5	Angeloylgomisin Q	3.51	553.2417	C_29_H_38_O_9_	30, 60, 120
P6	Gomisin G	4.08	537.2090	C_30_H_32_O_9_	30, 60, 120, 360, 480
P7	Gomisin K	4.19	403.2094	C_23_H_30_O_6_	30, 60, 120, 360
P8	Gomisin E	4.54	515.2264	C_28_H_34_O_9_	30, 60, 120, 360, 480
P9	Deoxyschizandrin ^a^	6.84	417.2258	C_24_H_32_O_6_	30, 60, 120, 360, 480
P10	Schisandrin B ^a^	10.21	401.1948	C_23_H_28_O_6_	30, 60, 120, 360, 480

^a^ Compounds were compared with the reference compounds.

**Table 2:** Summary of the lignan metabolites in *S. chinensis* in the plasma of dosed rats with Alzheimer’s disease.

Name	Metabolic Pathways	RT (min)	Measured Mass	Formula	Mass Error (ppm)	Time Points (min)	MS^2^
Schisandrin ^a^		2.17	433.2210	C_24_H_32_O_7_	−2.4	30, 60, 120	
M1	Hydroxylation+demethylation	1.46	457.1823	C_23_H_30_O_8_	−2	30, 60, 120, 360, 480	399, 385, 354, 367
M2	2 × Demethylation	1.48	427.1695	C_22_H_28_O_7_	1.48	60, 360	387, 385, 354
M3	Hydroxylation	1.7	449.2158	C_24_H_32_O_8_	−2.6	30, 60, 120, 360, 480	413, 359,383
M4	Demethylation	1.74	419.2054	C_23_H_30_O_7_	−2.4	30, 60, 120, 360	401, 373, 370, 359
M5	Phosphorylation	1.99	513.1912	C_24_H_33_O_10_P	5.5	360, 480	415, 384, 385,373
M6	2 × Demethylation+dehydration	5.2	387.1786	C_22_H_26_O_6_	−3.9	30, 60, 120, 360, 480	385, 354, 338, 323
Schisandrol B ^a^		2.54	439.1737	C_23_H_28_O_7_	2.2	30, 60, 120	
M7	2 × Demethylation	1.46	389.1594	C_21_H_24_O_7_	0.2	30, 60, 120, 360, 480	355, 322, 294
M8	Reduction+demethylation	1.48	427.1695	C_22_H_28_O_7_	−7.5	60, 360	387, 331, 345
M9 (Schisandrin)	Reduction+methylation	2.17	433.2210	C_24_H_32_O_7_	−2.4	30, 60, 120	415, 400, 385, 384, 373, 369, 359, 354, 353, 338, 322
M10	CH_2_O (cleavage)+dehydration	2.54	369.1673	C_22_H_24_O_5_	−6.4	30, 60, 120	353, 337, 264
M11	Reduction+dehydration	3.12	401.1923	C_23_H_28_O_6_	−9	360	370, 345, 359, 386
M12	Reduction + dehydration + demethylation	5.2	387.1786	C_22_H_26_O_6_	−4.1	30, 60, 120, 360, 480	331, 345
Angeloylgomisin Q		3.51	553.2417	C_29_H_38_O_9_	1.7	30, 60, 120	
M9 (Schisandrin)	C_5_H_6_O_2_ (cleavage)	2.17	433.2210	C_24_H_32_O_7_	−2.4	30, 60, 120	415, 400, 385, 384
M13	Demethylation	2.27	539.2239	C_28_H_36_O_9_	−2.2	30, 60, 120	417, 399, 389, 387, 369
Gomisin G		4.08	537.2090	C_30_H_32_O_9_	−5.4	30, 60, 120, 360, 480	
M14	C_7_H_4_O_2_(cleavage) + 2 × demethylation	1.46	389.1594	C_21_H_24_O_7_	0.2	30, 60, 120, 360, 480	371, 356, 340
M15	Demethylation	3.62	523.1932	C_29_H_30_O_9_	−5.7	30, 120, 360	401, 383, 369, 357,
Gomisin D		2.37	531.2228	C_28_H_34_O_10_	0.7	30, 60, 120, 360, 480	
M16	C_6_H_8_O_3_(cleavage) + reduction	1.48	427.1695	C_22_H_28_O_7_	−7.6	60, 360, 480	387, 359, 355
M17	Reduction	1.6	555.2165	C_28_H_36_O_10_	−6.5	30, 60, 120, 360, 480	485, 383, 352, 351, 341
M18	C_6_H_8_O_3_(cleavage)	1.63	425.1545	C_22_H_26_O_7_	−5.9	360, 480	385, 355, 353
M19	Demethylation	1.81	539.1884	C_27_H_32_O_10_	−0.5	360, 480	387, 368, 357
M20	C_6_H_8_O_2_(cleavage)	2.3	419.1666	C_22_H_26_O_8_	−8.3	30, 360	401, 383, 371, 351
M21	C_6_H_8_O_3_(cleavage) + dehydration	2.35	385.1629	C_22_H_24_O_6_	−4.3	30, 60, 120, 360, 480	355, 353
M22	C_6_H_8_O_2_(cleavage) + reduction	2.36	443.1699	C_22_H_28_O_8_	5.1	60, 120, 360, 480	403, 385, 357, 351
M23	Phosphorylation	3.62	611.1859	C_28_H_35_O_13_P	−4.8	60, 120, 360, 480	383, 371, 351
Gomisin E		4.54	515.2264	C_28_H_34_O_9_	−2.2	30, 60, 120, 360, 480	
M24	C_6_H_8_O_2_(cleavage)	1.73	403.1761	C_22_H_26_O_7_	2.5	120	385, 355, 354, 353, 343
M25	Decarboxylation	2.21	471.2388	C_27_H_34_O_7_	2.2	360, 480	385, 355, 354, 353, 343
M26	Reduction	2.27	539.2239	C_28_H_36_O_9_	−2.4	30, 60, 120	469, 355, 354, 353, 343, 329
M27	Hydration	4.77	555.2166	C_28_H_36_O_10_	−6.4	60, 360	385, 355, 354, 343
Benzoylgomisin H		3.12	523.2281	C_30_H_34_O_8_	−8.7	60, 120, 360	
M28	C_7_H_4_O(cleavage)	1.73	419.2061	C_23_H_30_O_7_	−0.6	30, 60, 120	401, 385, 316
Gomisin K		4.19	403.2094	C_23_H_30_O_6_	−5.3	30, 60, 120, 360	
M29	Hydroxylation + demethylation	1.48	427.1695	C_22_H_28_O_7_	−7.3	60	387, 372, 355, 333, 302
M30	Demethylation	1.72	389.1934	C_22_H_28_O_6_	−6.3	30, 60	374, 357, 333, 302
M31	Hydroxylation	1.73	419.2061	C_23_H_3_0O_7_	−0.8	30, 60, 120, 360	401, 386, 369, 333, 302
M32	2×Desaturation + acetyl cysteine conjugation	1.75	590.2380	C_28_H_41_NO_9_S	−2.6	30, 60, 120, 360, 480	389, 373, 359, 319
Deoxyschizandrin ^a^		6.84	417.2258	C_24_H_32_O_6_	−3.2	30, 60, 120, 360, 480	
M9 (Schisandrin)	Hydroxylation	2.18	433.2210	C_24_H_32_O_7_	−2.4	30, 60, 120	415, 400, 385, 384, 373, 369, 359, 354, 353, 338, 322
M28	Demethylation + hydroxylation	1.73	419.2061	C_23_H_30_O_7_	−0.6	30, 60, 120	401, 370, 369, 337
M33	Demethylation	4.65	403.2093	C_23_H_30_O_6_	−5.3	30, 60, 120, 360, 480	388, 385, 372, 371, 370, 339
M34	2 × Hydroxylation	1.7	449.2154	C_24_H_32_O_8_	−3.5	30, 60, 120, 360, 480	431, 413, 398, 383, 343, 316
M35	2 × Desaturation	3.48	413.1944	C_24_H_28_O_6_	−3.6	60, 120, 360, 480	398, 383, 382, 366, 347, 316
Schisandrin B ^a^		10.21	401.1948	C_23_H_28_O_6_	−2.7	30, 60, 120, 360, 480	
M36	Reduction + phosphorylation	1.68	483.1799	C_23_H_31_O_9_P	4.1	30, 60, 120, 360	387
M37	Demethylation + phosphorylation	1.81	467.1488	C_22_H_27_O_9_P	4.8	60	370, 371,300
M38 (Schisandrol B)	Hydroxylation	2.54	439.1737	C_23_H_28_O_7_	2.2	30, 60, 120	399, 384, 369, 368, 357, 353, 343, 341, 337, 338, 295
M39 (Deoxyschizandrin)	Reduction + methylation	6.84	417.2258	C_24_H_32_O_6_	−3.2	30, 60, 120, 360, 480	402, 386, 370, 371, 355, 347, 332, 316

^a^ Compounds were compared with the reference compounds.

**Table 3:** The relative potential targets induced by lignans in *S. chinensis*.

Target Gene	Pathway	Relative Target	Relative Pathway	Effective Constituents
AChE	Cholinergic synapse	APP	amyloid precursor protein metabolism	Gomisin K; Gomisin G
iNOS	Arginine and proline metabolism	Neurotransmitter; Calmodulin; IL	Nitric oxide anabolism; calmodulin binding; neurotransmitter metabolism; inflammatory response	Gomisin K
PTGS1	Arachidonic acid metabolism	PGs	Platelet activation; Cytochrome P450 - arranged by substrate type	Gomisin K
PTGS2	Arachidonic acid metabolism	PGs	inflammatory response; Cytokine signaling in immune system	Gomisin K; Benzoylgomisin H; Schisandrol B; Gomisin D
GSK3β	PI3K-Akt signaling pathway; Wnt signaling pathway	Tau; DA	MAPK signaling pathway; Dopaminergic synapse; neurofibrillary tangles	Gomisin K
TGFB1	MAPK signaling pathway	TNF-α	TNF signaling pathway	Schisandrin B
MAPK1	MAPK signaling pathway	Glu; ACh; 5-HT	Glutamatergic synapse; Cholinergic synapse; Serotonergic synapse	Schisandrin B
MAPK3	MAPK signaling pathway	Glu; ACh; 5-HT	Glutamatergic synapse; Cholinergic synapse; Serotonergic synapse	Schisandrin B
TBXAS1	Cytochrome P450 - arranged by substrate type	AA	Arachidonic acid metabolism; Platelet activation	Schisandrin B
CYP3A4	Drug metabolism - cytochrome P450	Cholesterol; LA; Retinoate	Steroid hormone biosynthesis; Linoleic acid metabolism; Retinol metabolism	Schisandrin B
PPARG	PPAR signaling pathway	9-CRA	Lipid metabolism; Antioxidant system	Gomisin K
TOP2	Metabolism of proteins	/	Cell cycle	Gomisin K; Angeloylgomisin Q; Benzoylgomisin H; Schisandrol B; Gomisin D; Gomisin G
Mir34a	MicroRNAs in cancer	Cyclin-dependent kinase 6	Cell cycle	Schisandrin B
CCND1	Cyclins and cell cycle regulation	/	/	Schisandrin B
GSR	Glutathione metabolism	GSH	Glutathione metabolism; Antioxidant system	Schisandrin B
NFE2L2	Protein processing in endoplasmic reticulum	HMOX1	Porphyrin and chlorophyll metabolism	Schisandrin B; Deoxyschizandrin
HMOX1	Porphyrin and chlorophyll metabolism	APP; IL-10; NFE2L2	amyloid precursor protein metabolism; Protein processing in endoplasmic reticulum;Cytokine signaling in immune system	Schisandrin

**Table 4:** Changes in the main groups in the metabolic pathways of lignans from *S. chinensis.*

Description	Formula	Delta Mass (Da)	Classifier
Parent	/	/	/
Methylation	+CH_2_	14.0157	Phase II
Demethylation	–CH_2_	−14.0157	Phase I
Reduction	+H_2_	2.0157	Phase I
Desaturation	–H_2_	−2.0157	Phase I
2×Desaturation	–H_4_	−4.0313	Phase I
Hydroxylation	+O	15.9949	Phase I
2×Hydroxylation	+O_2_	31.9898	Phase I
3×Hydroxylation	+O_3_	47.9847	Phase I
Nitro reduction	–O_2_+H_2_	−29.9742	Phase I
Dehydration	–H_2_O	−18.0106	Phase I, II
Hydration	+H_2_O	18.0106	Phase I
Dihydrodiol formation	+H_2_O_2_	34.0055	Phase I
Decarbonylation	–-CO	−27.9949	Phase I
Formylation	+CO	27.9949	Phase II
Decarboxylation	–COO	−43.9898	Phase I
Phosphorylation	+HPO_3_	79.9663	Phase II
Acetyl cysteine conjugation	+C_5_H_7_NO_3_S	161.0147	Phase II
2 × Glucuronide conjugation	+C_12_H_16_O_12_	352.0642	Phase II
Glucuronidation	+C_6_H_8_O_6_	176.0321	Phase II

**Table 5:** Multiple reaction monitoring-optimized parameters for quantitation in rat plasma.

Mode	Compounds	Capillary Voltage (kV)	Nebulizer Gas (L/h)	Desolvation Gas (L/h)	Cone Voltage (V)	Quantitation Transition (*m*/*z*, Collision (eV)^1^)	Confirmation Transition (*m*/*z*, Collision (eV) ^1^)
ESI^+^	Gly	2.5	50	800	14	75.97 > 30.19 (8)	75.97 > 48.14 (6)
	Asp	2.5	50	800	12	133.97 > 74.03 (14)	133.97 > 88.07 (10)
	Glu	2.5	50	800	14	147.97 > 84.11 (16)	147.97 > 130.03 (8)
	GABA	2.5	50	800	20	103.97 > 86.99 (10)	103.97 > 68.03 (14)
	NE	2.5	50	800	6	169.97 > 152.04 (8)	169.97 > 107.3 (18)
	ACh	2.5	50	800	22	146.03 > 87.04 (12)	146.01 > 60.11 (10)
	DA	2.5	50	800	12	153.97 > 137 (10)	153.97 > 91.08 (22)
	5-HT	2.5	50	800	10	176.97 > 160.02 (8)	176.97 > 132.06 (20)
	9-CRA	3	40	500	20	301.29 > 123.17 (22)	301.29 > 161.40 (22)
ESI^−^	AA	2.6	50	650	32	303.35 > 259.29 (12)	303.35 > 205.21 (16)
	LA	2.8	40	500	36	279.35 > 261.23 (18)	279.35 > 59.13 (18)
	cholesterol sulfate	2.6	50	650	62	465.48 > 97.03 (36)	465.48 > 80.00 (80)

## Supplementary Materials

The following are available online, Figure S1: Total ion current chromatogram of lignans isolated from *S. chinensis* in the positive mode, Figure S2: Extracted ion chromatograph and MS/MS spectrum of schisandrin in purified lignans (a) and the standard (b), Figure S3: MS/MS spectrum of 10 prototype compounds in dosed plasma (A), purified lignans, (B) and the standard (C), Table S1: Identification of compounds detected in the positive mode.
