# Supplementary material for: Systematically Characterize the Anti-Alzheimer’s Disease Mechanism of Lignans from S. chinensis Based on In-Vivo Ingredient Analysis and Target-Network Pharmacology Strategy by UHPLC–Q-TOF-MS
Source: Molecules. 2019 Mar 27;24(7):1203. doi: 10.3390/molecules24071203 (PMC6480032; doi:10.3390/molecules24071203)
Supplement: Supplementary file 1 [file molecules-24-01203-s001.pdf]

Systematically characterize the anti-Alzheimer's disease mechanism of lignans in *S. chinensis* based on in-vivo ingredients analysis and target-network pharmacology strategy by UHPLC-Q-TOF-MS

Mengying Wei [1,2], Yuanyuan Liu [1], Zifeng Pi [2], Shizhe Li [3], Mingxin Hu [1], Yang He [4], Kexin Yue [1], Tianshu Liu [1], Zhiqiang Liu [2], Fengrui Song [2], Zhongying Liu [1,*]

[1] School of Pharmaceutical Sciences, Jilin University, 1266 Fujin Road, Changchun, 130021,China
[2] National Center for Mass Spectrometry in Changchun, Jilin Province Key Laboratory of Chinese Medicine Chemistry and Mass Spectrometry, Changchun Institute of Applied Chemistry, Chinese Academy of Sciences, Changchun 130022, China
[3] Guangdong Univ Technol, Inst Biomed & Pharmaceut Sci, Guangzhou 510006, Guangdong, Peoples R China
[4] Zhuhai College of Jilin University, Zhuhai 519041, China
* Correspondence: liuzy@jlu.edu.cn; Tel.: +86 431 85619704 (Z.L.)

1 Preparation of lignans purification isolated from *S. chinensis*

The 50 g crude herbs were soaked overnight with 750 mL water. And then the extraction method of drug sediments were described below: 80% ethanol of extraction solvent, homogenate extraction time and voltage of 100 s and100 v, liquid to solid ratio of 19 : 1. This supernatant was purified with AB-8 macroreticular adsorption resin to get the lignans purification from the extrait of *S. chinensis*. The purity of lignans from *S. chinensis* could reach to more than 84%.

2 Identification of lignans isolated from *S. chinensis* in purification

The column temperature was kept at 40 °C and the injection volume was 5 μL. The mobile phase consisted of 0.1 % formic acid (v/v) (A) and methyl alcohol (B) at a flow rate of 0.35 mL/min. The gradient elution in positive mode was performed as follows: 5 % - 55 % B at 0 - 0.5 min, 55 % - 88 % B at 0.5 - 8 min, 88 % - 100 % B at 8 - 10 min, 100 % - 5 % B at 10 - 10.5 min, and 5 % B at 10.5 - 15 min. The electrospray ionization (ESI) source with a scanning mass-to-charge (m/z) range from 50 to 1000 Da was used for the experiment. The source temperature was 120 °C. Nitrogen was used as cone and desolvation gas. The flow rates were 50 L/h and 700 L/h, respectively and the solvent temperature was 350 °C. The cone and extraction cone voltages were 40 V and 5.0 V, respectively. The capillary voltage was set to 3.0 kV. Quality standard curves were established using sodium formate. Leucine enkephalin was used as reference mass. The MSE data acquisition was performed using He as collision gas respectively with a low collision energy of 5 eV and a high collision energy of 10 - 30 eV. The database of lignans in *S. chinensis* based on mass spectrum database (PubMed, Mass Bank, Chemspider, and METLIN) and references was built in UNIFI TM (Waters Corp. Milford, MA, USA). The database of lignans in *S. chinensis* included compound name, molecular formula, structural formula and fragment ion. Then MassFragment TM and UNIFI TM were used to analysis raw data of lignans purification isolated from *S. chinensis*.

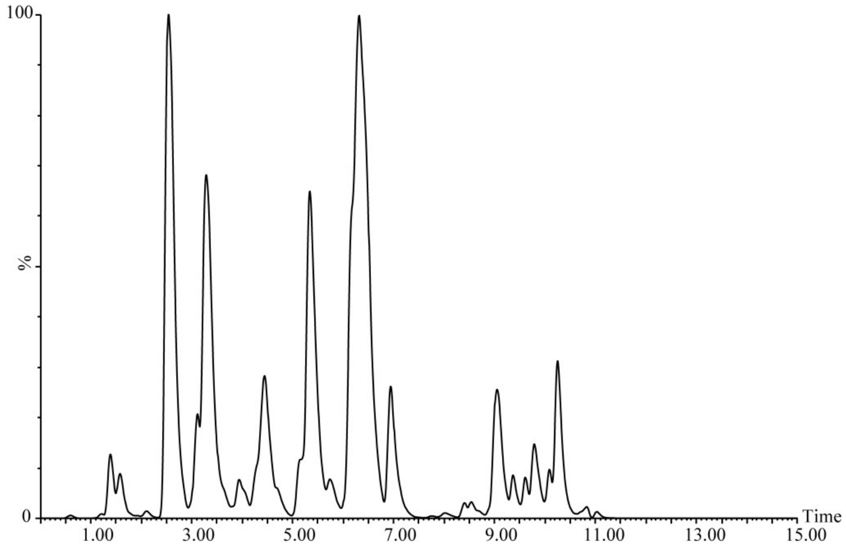

Figure S1. Total ion current chromatogram of lignans isolated from *S. chinensis* in positive mode

The base peak ion chromatogram (BPI) of lignans isolated from *S. chinensis* was showed in Figure S1. Those unknown compounds were identified using MS or MS/MS spectral databases. And some of those were also compared with reference standards. What follows was the description of the identification procedure taking schisandrin as examples. The ion exhibited a retention time of 2.57 min and the [M + H] + ion at m/z 433.2043 in positive mode. Via searching UNIFI databases, the compound may be identified as schisandrin. Then we confirmed that its retention times and accurate masses were consistent with reference standards. In MS/MS analysis, fragment ions of schisandrin in lignans purification were also the same as the reference standards (Figure S2). Finally the fragment ions were identified to be schisandrin. And then fourteen lignans were identified which details were listed in Table S1, including schisandrin, gomisin D, gomisin J, benzoylgomisin H, angeloylgomisin Q, schisandrol B, gomisin G, schisantherin A, gomisin K, tigloylgomisin H, gomisin E, deoxyschizandrin, schisandrin B amd schisandrin C.

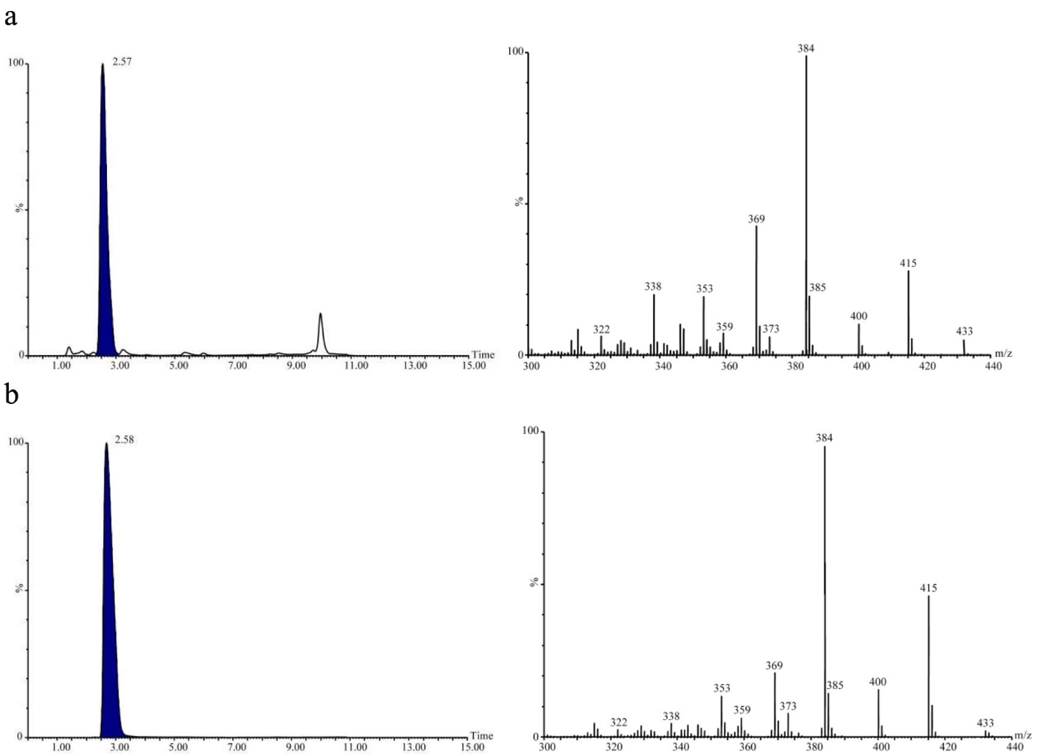

Figure S2. The extracted ion chromatograph and MS/MS spectrum of schisandrin in lignans purification (a) and standard (b)

Table S1. Identification of compounds detected in positive mode

Compounds	RT (min)	Measured mass	Formlar	Mass error(ppm)	Adducts	MS^2
Schisandrin [a]	2.57	455.2043	$C_{24}H_{32}O_7$	0.7	+Na, +H	415, 400, 385, 384, 373, 369, 359, 354, 353, 338, 322
Gomisin D	2.97	553.2061	$C_{28}H_{34}O_{10}$	3	+Na, +H	485, 401, 383, 371, 369, 353, 351
Gomisin J	3.13	389.1960	$C_{22}H_{28}O_6$	0.5	+H, +Na	374, 358, 342, 319, 288
Benzoylgomisin H	3.34	523.2305	$C_{30}H_{34}O_8$	-4.1	+H	505, 401
Angeloylgomisin Q	3.43	553.2403	$C_{29}H_{38}O_9$	-0.9	+Na	431, 413, 401, 399, 389, 387, 357
Schisandrol B [a]	3.45	439.1907	$C_{23}H_{28}O_7$	1	+Na, +H	399, 384, 369, 368, 357, 353, 343, 341, 337, 338, 295
Gomisin G	4.37	537.2109	$C_{30}H_{32}O_9$	-1.9	+H, +Na	415, 397, 384, 373, 371, 356, 341, 340
Schisantherin A [a]	4.52	537.5765	$C_{30}H_{32}O_9$	0.4	+H, +Na	415, 397, 385, 373, 371, 367, 356, 340, 325, 310, 297, 295, 267
Gomisin K	4.71	403.2120	$C_{23}H_{30}O_6$	1.1	+H, +Na	388, 371, 340, 333, 302, 301
Tigloylgomisin H	4.76	523.2296	$C_{28}H_{36}O_8$	-1.2	+Na	483, 401, 357
Schisantherin B [a]	4.84	537.2098	$C_{28}H_{34}O_9$	0.6	+Na	415, 397, 385, 371
Gomisin E	5.17	515.2288	$C_{28}H_{34}O_9$	2.3	+H, +Na	469, 385, 367, 355, 354, 353, 343, 337, 329, 323
Deoxyschizandrin [a]	5.36	417.2276	$C_{24}H_{32}O_6$	1	+H, +Na	402, 386, 370, 371, 355, 347, 332, 316
Schisandrin B [a]	6.33	401.1962	$C_{23}H_{28}O_6$	0.8	+H, +Na	386, 371, 370, 355, 354, 340, 339, 331, 329, 316, 300, 285, 273, 270
Schisandrin C [a]	6.96	385.1642	$C_{22}H_{24}O_6$	-0.8	+H, +Na	370, 355, 325, 315, 300, 285, 257, 227

[a] Compounds were compared with reference compounds.

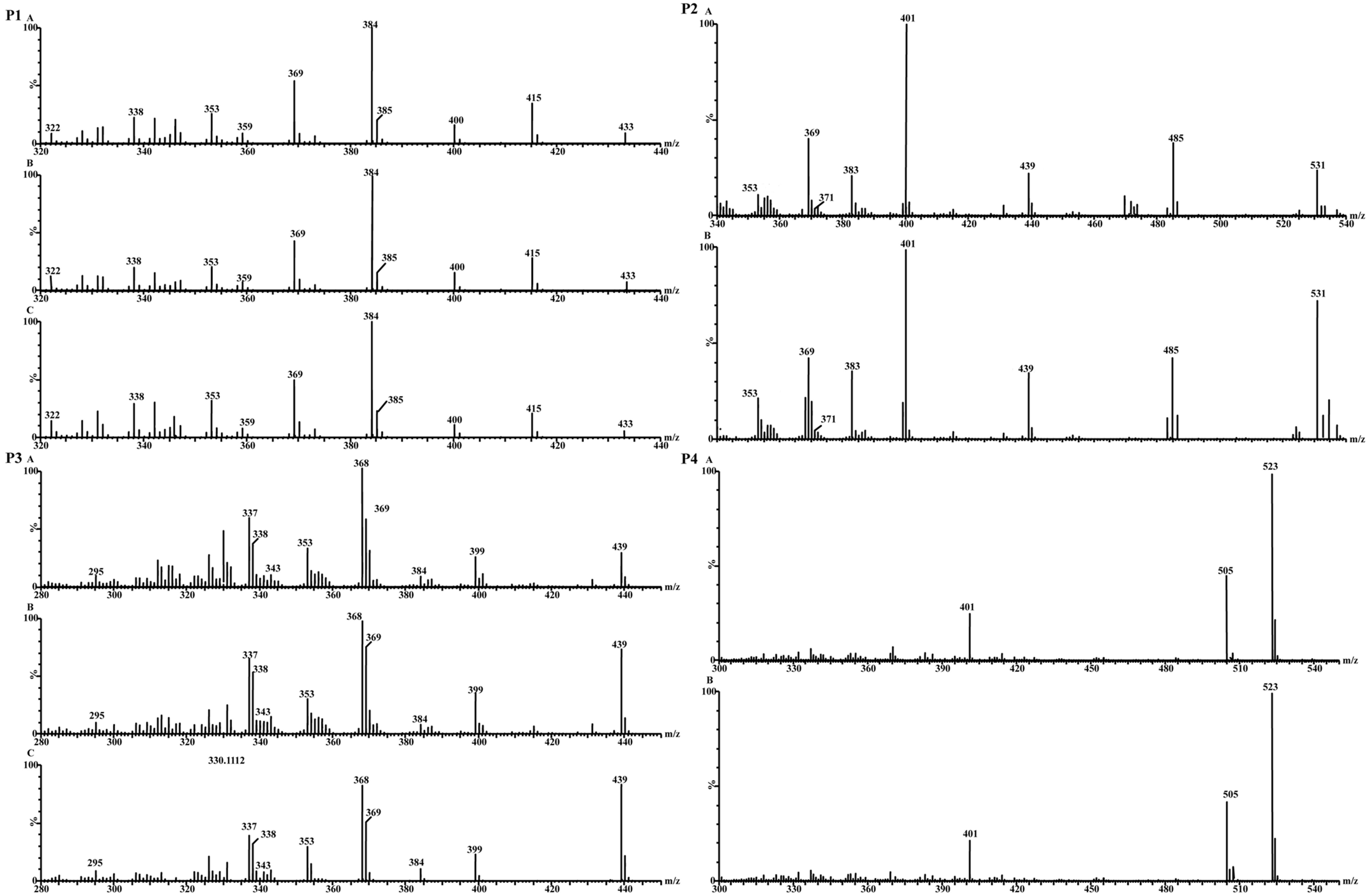
P1
A
384
369
338
353
359
322
385
400
415
433
m/z
%
B
C
P2
401
369
383
353
371
439
485
531
P3
368
369
337
338
353
343
295
384
399
439
330.1112
P4
523
505
401

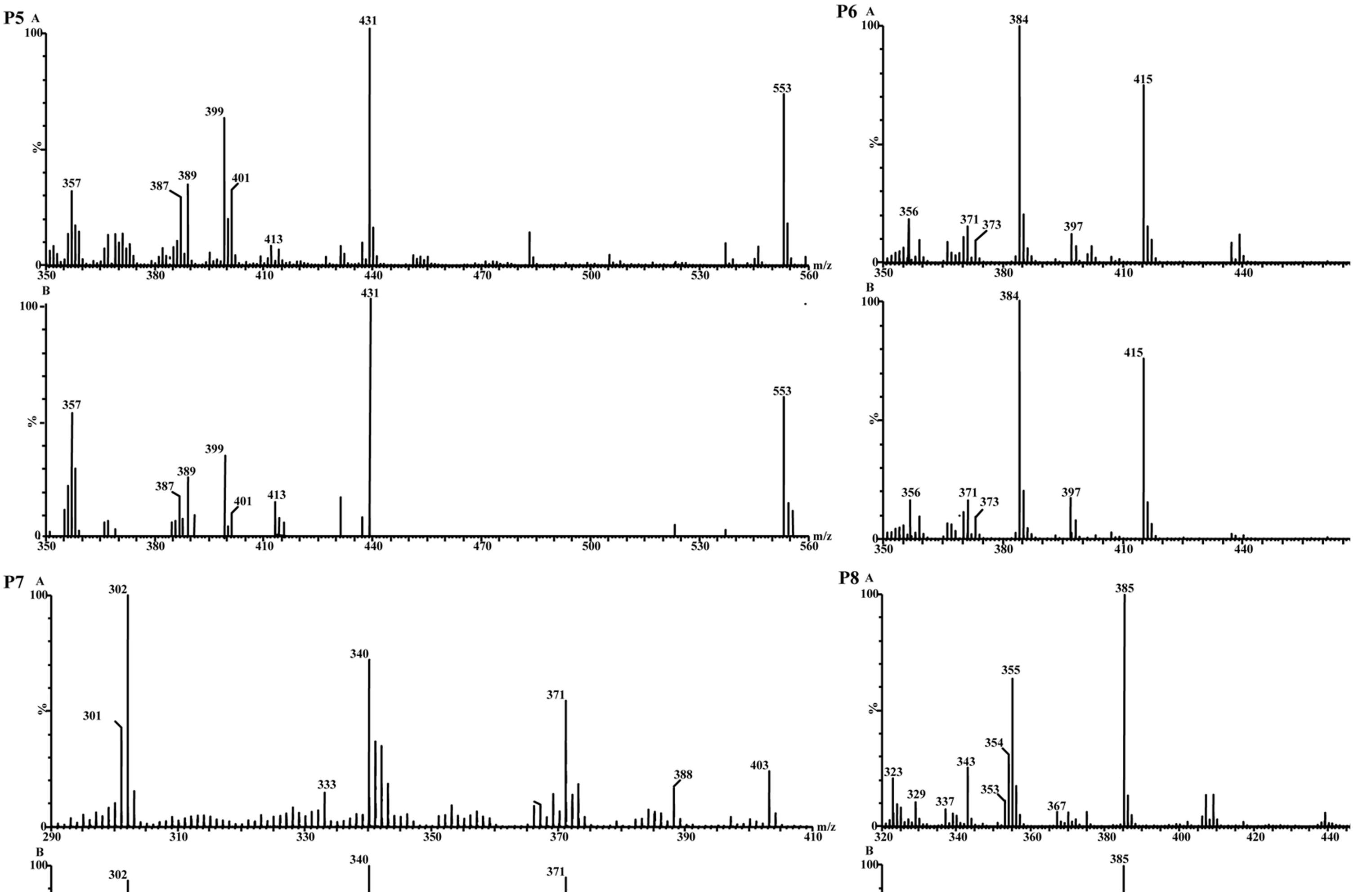
P5
A
431
553
399
357
387
389
401
413
m/z
B
431
553
357
399
387
389
401
413
P6
A
384
415
356
371
373
397
B
384
415
356
371
373
397
P7
A
302
340
301
371
333
388
403
m/z
B
302
340
371
P8
A
385
355
354
343
323
353
329
337
367
B
385
%

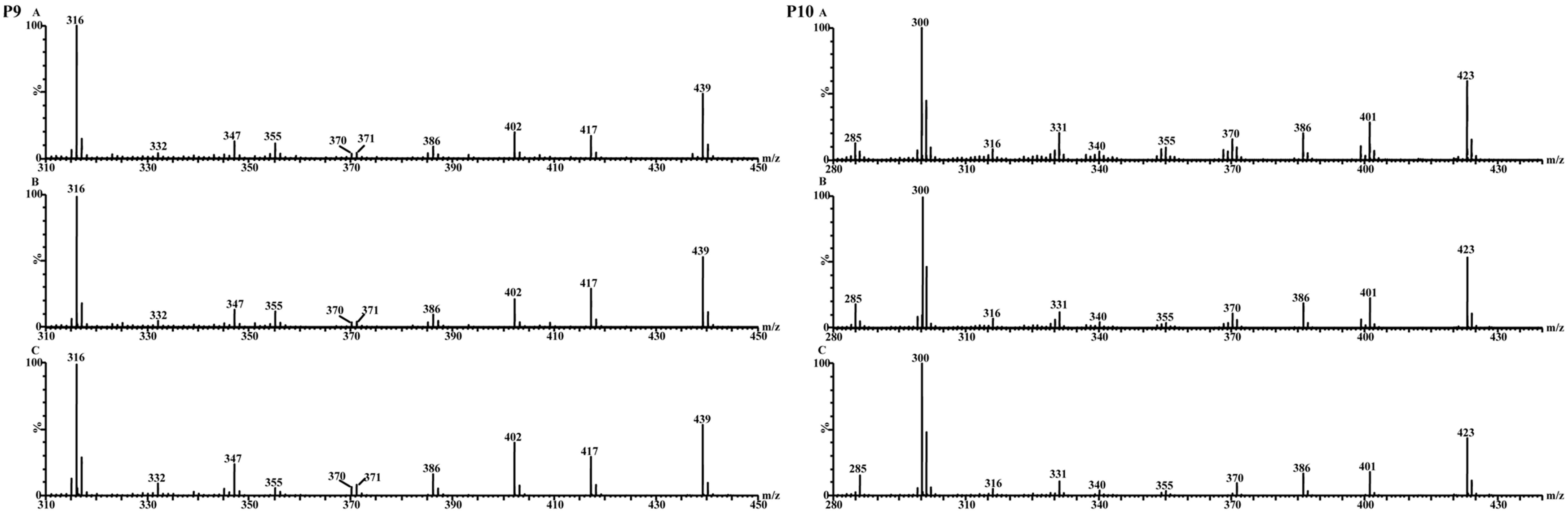

Figure S3. The MS/MS spectrum of 10 prototype compounds in dosed plasma (A), lignans purification (B) and standard (C)
